# Physical rehabilitation techniques for spinal cord injury: A scoping review

**DOI:** 10.4102/ajod.v15i0.1869

**Published:** 2026-05-07

**Authors:** Candace Vermaak, Danet Cloete, Jared Kunene, Megan Webb, Inge von Wielligh, Vuyelwa Radebe

**Affiliations:** 1 Department of Exercise, Sport and Lifestyle Medicine, Faculty of Medicine and Health Sciences, Stellenbosch University, Cape Town, South Africa

**Keywords:** spinal cord injury, physical activity, sport, rehabilitation, exercise

## Abstract

**Background:**

Spinal cord injury (SCI) is a life-changing condition resulting in disability, with motor and sensory impairments that impact multiple areas of life and reduce quality of life. Physical rehabilitation can address these limitations, but there is a need to evaluate which interventions are most effective and the outcomes they produce.

**Objectives:**

To evaluate the effectiveness of physical rehabilitation interventions for individuals with complete and incomplete SCI at levels C5–T12, and to inform clinical practice.

**Method:**

This review followed the Preferred Reporting Items for Systematic Reviews and Meta-Analyses extension for scoping reviews. A comprehensive search of PubMed, Scopus and EBSCOhost identified peer-reviewed studies published between 2013 and 2024.

**Results:**

Sixteen studies (*n* = 327) were included. Effective interventions included functional electrical stimulation, robotic and exoskeleton-assisted training, gait training, upper extremity exercise, balance training, and corporal suspension and pendulum exercises. Reported outcomes included improvements in aerobic capacity, muscle adaptations, gait parameters, cardiopulmonary function, functional capacity and secondary complications.

**Conclusion:**

Physical rehabilitation plays a key role in improving functional outcomes in individuals with SCI. However, no single intervention addresses all aspects of recovery, highlighting the need for an individualised approach.

**Contribution:**

This review demonstrates that a range of exercise-based rehabilitation strategies can enhance functional outcomes in individuals with SCI.

## Introduction

A spinal cord injury (SCI) is a life-altering injury leading to permanent disability and reduced quality of life (Gaspar et al. 2019). A SCI involves damage to the nerve fibres extending from the lower part of the brain through the vertebral column, disrupting the structural and functional connections between the spinal cord and the higher brain. Depending on the extent and location of the lesion, someone with SCI may experience a partial or complete loss of motor, sensory and autonomic function below the level of injury (Alizadeh, Dyck & Karimi-Abdolrezaee 2019). A SCI can be categorised as either incomplete or complete (Alizadeh et al. 2019). An incomplete SCI leads to partial loss of function below the level of injury, with some motor and sensory function left due to some nerve fibres’ axons remaining intact (Anjum et al. 2020). A complete SCI is more severe, with neither motor nor sensory function remaining, leading to total loss of function below the level of injury (Alizadeh et al. 2019). Cervical injuries often result in tetraplegia (quadriplegia), resulting in loss of all four limbs’ sensory or motor function, either completely or partially, whereas thoracic injuries can lead to paraplegia, affecting the trunk and lower limb function (Alizadeh et al. 2019). A SCI presents itself with secondary health conditions and associated complications, including pain, spasticity, urinary tract infections, loss of bowel and bladder control, respiratory and circulatory dysfunction, autonomic dysreflexia and pressure ulcers (Sargent et al. 2023). Because of the various barriers (personal, environmental and task) faced by individuals with SCI, they often adopt a sedentary lifestyle, resulting in significantly decreased physical activity levels (Maher, McMillan & Nash; Vermaak et al. 2022). Notable consequences of reduced physical activity include musculoskeletal changes such as muscle atrophy, osteopenia or osteoporosis, hypertonia and limited joint mobility (Gaspar et al. 2019). Secondary health and associated conditions reduce the ability of individuals with SCI to retain their independence, health and well-being. These conditions negatively impact quality of life and ability to participate (Richardson et al. 2021). Rehabilitation for individuals with SCIs strives to maximise functional recovery, improve quality of life and promote independence (Farahani et al. 2021). Exercise as a rehabilitation method offers multiple health benefits, including enhanced cardiovascular fitness, improved respiratory function, increased muscle strength and psychological benefits, such as improved mental health (Akkurt et al. 2017). Exercise is known to improve autonomy in activities of daily living (ADL), prevent and manage secondary and associated conditions, and promote an active lifestyle in the SCI population (Akkurt et al. 2017). The wide variety of SCI characteristics, such as the level and completeness of injury, necessitates tailored rehabilitation programmes to meet the particular needs of each individual. Furthermore, advancements in rehabilitation techniques continue to evolve, making it important for physical rehabilitation therapists to stay informed about the latest and most effective rehabilitation interventions for individuals with SCI. The objective of this scoping review is to provide a comprehensive review that synthesises the current evidence on rehabilitation interventions for SCI rehabilitation, highlighting best practices and identifying gaps in the literature that require further research. This article aims to contribute to existing information on effective rehabilitation interventions for individuals with SCIs at levels C5 to T12.

## Methods

### Search strategy

A comprehensive review of the existing literature was conducted using the Preferred Reporting Items for Systematic reviews and Meta-Analyses extension for Scoping reviews (PRISMA-ScR). An electronic literature search was conducted using three databases: PubMed, Scopus and EBSCOhost. The search terms used in this scoping were (‘spinal cord injury’) AND ([‘exercise’ AND ‘intervention’] OR [‘physical’ AND ‘rehabilitation’]) NOT (‘systematic review’). Additionally, studies were required to be available in English, in full-text, in peer-reviewed scientific journals, be human-based and be published between January 2013 and January 2024.

### Study selection

Database searches were conducted by three reviewers (Danet Cloete, Jared Kunene and Megan Webb), independently. Three reviewers (Megan Webb, Danet Cloete and Inge von Wielligh) removed duplicate studies through a systematic process using Excel and screened the titles and abstracts for eligibility. Following the screening phase, two reviewers (Inge von Wielligh and Megan Webb) independently evaluated full-text studies for inclusion. Any discrepancies were addressed through discussion and consensus, with the aid of a third reviewer (Danet Cloete). Studies were included if they adhered to the following criteria: (1) the injury being treated is a SCI; (2) the study reported on any form of exercise intervention or physical rehabilitation; (3) the study focused on SCI levels C5–T12; and (4) the study participants were older than 18 years of age. Studies were excluded if they: (1) were animal-based; (2) were published as a review, case study, pilot study or discussion paper; and (3) the findings of the study were not applicable to the scope of physical rehabilitation therapists.

### Data extraction

Three independent reviewers used Microsoft Excel to compile a data extraction table (Danet Cloete, Inge von Wielligh and Megan Webb). The following data were extracted from each study: (1) publication year and author(s) of the study; (2) number of participants; (3) participant demographics, including age, level of injury and time since injury occurrence; (4) intervention type, length and frequency; (5) structure of session; and (6) outcome measures of the intervention.

### Ethical considerations

An application for full ethical approval was made to the Health Research Ethics Committee, Stellenbosch University, and ethics consent was received on 11 September 2025 (Ref no. X25/08/024). The Health Research Ethics Committee issued an ethics waiver for the study because the study did not involve human participants.

## Review findings

### Literature search and study selection

**FIGURE 1:**
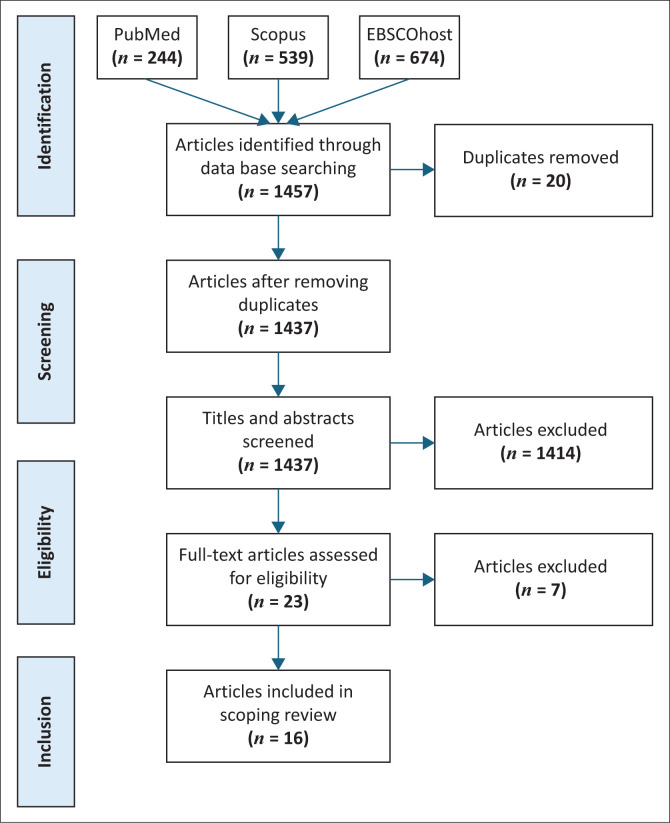
Preferred Reporting Items for Systematic reviews and Meta-Analyses flow-chart.

Database search yielded 1457 articles. After removing 20 duplicate studies, 1437 studies were screened for eligibility based on title and abstract. This left 23 articles for full-text screening, of which seven did not meet the inclusion criteria, leaving 16 studies for this scoping review, as shown in Figure 1.

### Participant characteristics

This review includes 16 studies, with a total of 327 participants. Participants in the included studies were heterogeneous with respect to sex, age and completeness of the injury. Participants’ ages ranged from 18 years to 70 years, with an injury level between C5 and T12. The time since injury among participants ranged from acute (< 6 months) to chronic (> 6 years).

### Intervention characteristics

This review focused on the types of interventions utilised and the specific outcomes evaluated through each intervention. The following interventions were examined: functional electrical stimulation (FES), robotic and exoskeleton training, gait training (excluding robotic or exoskeleton assistance), upper extremity exercise training, balance training, and corporal suspension and pendulum exercises. Each intervention and its associated functional outcomes are detailed below.

### Functional electrical stimulation

Three out of the 16 studies utilised FES as part of their training modalities (Lambach et al. 2020; Ryan et al. 2013; Taylor et al. 2014). Lambach et al. (2020) paired FES with rowing as an intervention to investigate the effect on bone mineral density (BMD). In comparison to Ryan et al. (2013), who utilised FES with resistance training to investigate the impact on glucose tolerance and insulin resistance, modifications to muscle mass, composition, and metabolism in muscles below the level of injury. Lastly, Taylor et al. (2014) reported on a combination of FES rowing and FES resistance training to determine the effect on aerobic capacity.

### Robotic and exoskeleton training

Three studies examined robot-assisted or exoskeleton training (Gagnon et al. 2018; Martinez et al. 2018; Park et al. 2021). Martinez et al. (2018) used multimodal exercise training combined with robot-assisted treadmill training in their intervention to investigate the effects of multimodal training on lower extremity motor scores (LEMSs) and corticospinal neurotransmission compared with treadmill training alone. Two studies implemented exoskeleton-assisted overground walking training in their exercise sessions (Gagnon et al. 2018; Park et al. 2021). Gagnon et al. (2018) focused on gait parameters such as the number of steps taken, as well as gait speed and duration, whereas Park et al. (2021) focused on both gait parameters and aerobic capacity as outcome measures.

### Gait training (without exoskeleton or robotic assistance)

Four studies investigated gait training without robotic assistance or the use of an exoskeleton (Amatachaya et al. 2019; Echemendía del Valle et al. 2023; Nithiatthawanon et al. 2020; Pramodhyakul et al. 2016). Nithiatthawanon et al. (2020) evaluated functional capacity and mobility with and without overground walking, combined with external feedback. Amatachaya et al. (2019) examined the effects of reported dual and single-task obstacle-walking training on the cognitive and functional abilities of long-term ambulatory individuals with SCI. Pramodhyakul et al. (2016) investigated the effect of gait training, with or without visuotemporal cues, on various gait parameters, and Echemendía del Valle et al. (2023) focused on the effects of a gait training programme incorporating upper limb and trunk strength training on motor function and mobility.

### Upper extremity exercise training

Four out of the 16 studies reported on the use of upper extremity exercise training (Bresnahan et al. 2019; Ilyas et al. 2023; Kesiktaş et al. 2021; Kim et al. 2015). Kim et al. (2015) used indoor hand-bike training to evaluate its effects on muscle strength, aerobic capacity and insulin sensitivity. Kesiktaş et al. (2021) focused on home-based upper extremity circuit training exercises and the effect thereof on aerobic capacity. The other two studies included upper extremity ergometry training (Bresnahan et al. 2019; Ilyas et al. 2023). Ilyas et al. (2023) combined ergometry with conventional treatment to assess the effect on pulmonary functioning among individuals with SCI, and Bresnahan et al. (2019) evaluated the impact on aerobic capacity.

### Balance training

One study (Khurana, Walia & Noohu 2017) compared virtual reality game-based balance training and real-world, task-specific balance training with conventional therapy. Khurana et al. (2017) evaluated whether these modalities would lead to improvements in sitting balance and the capability to function among individuals with paraplegia.

### Corporal suspension and pendulum exercises

One out of the 16 studies investigated the impact of body suspension and pendulum exercises on neuromuscular function and functional capacity in individuals with thoracic SCI (Frison et al. 2019).

### Outcomes: Aerobic capacity

Five out of the 16 articles investigated aerobic capacity as an outcome (Bresnahan et al. 2019; Kesiktaş et al. 2021; Kim et al. 2015; Park et al. 2021; Taylor et al. 2014). Three studies which included indoor hand-bike training, home-based upper extremity circuit training exercises and upper extremity ergometric training reported significant increases in maximal oxygen consumption (VO_2_max), with *p*-values of 0.001, 0.012 and 0.046, respectively (Bresnahan et al. 2019; Kesiktaş et al. 2021; Kim et al. 2015). One of the studies that focused on exoskeleton-assisted walking showed no significant changes in VO_2_-rest, VO_2_-peak, or VO_2_-average (Park et al. 2021). However, one study that combined FES rowing with FES resistance training reported significant improvements in both peak aerobic capacity and peak minute ventilation from pre- to post-training, with *p*-values of 0.02 and 0.01, respectively (Taylor et al. 2014). The absence of significant changes in aerobic capacity following exoskeleton-assisted walking suggests that the type of exercise modality used plays a crucial role in achieving these outcomes (Park et al. 2021).

Individuals with an SCI typically have low levels of aerobic fitness because of limited movement capacity, which results in deconditioning and immobility, as well as the effects of the injury on the body’s response to exercise (Hodgkiss et al. 2023). Improving aerobic capacity will improve cardiovascular health, reducing the risk of heart disease, which is elevated in this population due to reduced physical activity and associated autonomic dysfunction (Hodgkiss et al. 2023). Improved aerobic capacity also allows for greater functional capacity and independence by making daily tasks less exhausting and improves metabolic health by enhancing insulin sensitivity and glucose metabolism, thus decreasing the risk of metabolic syndrome and diabetes (Hodgkiss et al. 2023). Regular aerobic exercise also contributes to better overall well-being and quality of life for individuals with an SCI (Hodgkiss et al. 2023).

### Cardiopulmonary function

One of the 16 studies reported cardiopulmonary function as an outcome (Ilyas et al. 2023). Ergometry training of the upper extremities, combined with conventional therapy, showed significant improvements in forced expiratory volume in one second (FEV1) and peak expiratory flow (PEF), with *p*-values of 0.008 and 0.001, respectively (Ilyas et al. 2023). These measures are crucial indicators of respiratory health, and given that respiratory complications are a primary cause of mortality and morbidity among individuals with SCI, enhancing these pulmonary functions through targeted interventions can significantly influence their overall health and quality of life (Berlowitz, Wadsworth & Ross 2016). Upper limb ergometer training combined with conventional therapy, including deep breathing, assistive coughing, sustained stretching, range of motion (ROM) exercises, tilt table standing and functional mobility exercises, also showed significant improvement in forced vital capacity (FVC) compared to conventional therapy alone (*p* = 0.003) (Ilyas et al. 2023).

Forced vital capacity is essential for maintaining adequate ventilation and preventing respiratory complications (Berlowitz et al. 2016). The significant improvement in FVC suggests that this combined intervention can more effectively address the respiratory limitations associated with SCI, leading to improved functional outcomes and reduced secondary complications (Berlowitz et al. 2016).

### Muscular adaptation

Three out of the 16 studies investigated the effects on muscular adaptations in individuals with SCI (Frison et al. 2019; Kim et al. 2015; Ryan et al. 2013). One study reported that home-based FES resistance exercise improved mitochondrial function in skeletal muscle and increased quadriceps muscle volume (Ryan et al. 2013). Kim et al. (2015) showed that indoor hand-bike training increased strength in shoulder extension and flexion, elbow extension and flexion, and shoulder adduction and abduction, along with an increase in the average lean muscle mass. The third study reported that body suspension and pendulum exercises using the CHORDATA^®^ method led to improvements in trunk flexion and extension torque as well as increased thickness of the rectus abdominis, longissimus, external oblique and internal oblique muscles, but no increased thickness of the right multifidus muscle as measured by ultrasound (Frison et al. 2019). Improvements in muscle volume and increased skeletal muscle mitochondrial function will allow for greater mobility and endurance in these individuals (Gorgey & Dudley 2007). The lack of increased thickness in the right multifidus muscle highlights the need for targeted interventions addressing spinal instability (Frison et al. 2019).

Studies highlight how exercise training may lead to structural and functional adaptations in skeletal muscles. Exercise may aid the activation of satellite cells and the regeneration of muscle fibres, resulting in improved muscle function, which is crucial after a SCI (Santos et al. 2022). For ADL, such as getting onto their wheelchairs, this population needs muscle power (Santos et al. 2022). Their arms need to be strong enough for activities like cleaning, self-care and transfers. Insufficient upper extremity strength can lead to pressure ulcers from prolonged wheelchair sitting, particularly when the patient is unable to adjust their posture (Santos et al. 2022). The mobility, risk of falling and functional capacity of individuals with SCI are directly impacted by muscle strength and adaptations (Santos et al. 2022).

### Gait parameters

Four of the five studies demonstrated improvements in walk test performance. Two studies reported improvements in the 10-metre walk test (10MWT), one study reported improvements in the 6-minute walk test (6MWT), and one study reported improvements in both the 6MWT and 10MWT. Dual-task and single-task obstacle walking training (Amatachaya et al. 2019), lower limb loading exercises, and bodyweight shifting, both with and without overground walking and external feedback (Nithiatthawanon et al. 2020), have been shown to improve 10MWT measurements. However, the group that received overground gait training along with external feedback showed more substantial improvements compared to the group that did not undergo these interventions (Nithiatthawanon et al. 2020). This is explained by external feedback, which provides additional information that helps them control and modify their motions in accordance with the demands of the task (Nithiatthawanon et al. 2020). By providing participants with the necessary information, they were able to maximise use of the stance limb, which minimised the taught non-use of the training leg or the injured lower limbs and decreased their dependence on their upper limbs (Nithiatthawanon et al. 2020). It has been reported that exoskeleton-assisted walking improved 6MWT walking distance from pre- to post-intervention (Park et al. 2021). Participants who received walking training with visuotemporal cues showed a much greater improvement in both the 10MWT and 6MWT than those who did not receive visuotemporal cues (Pramodhyakul et al. 2016). Additionally, one study reported that a robotic exoskeleton combined with a locomotor training programme demonstrated improvements in standing time, walking time, the number of steps taken per session and walking speed, with a significant decrease in the amount of therapist support needed (Gagnon et al. 2018). These improvements were likely because of increased motor learning and neural plasticity, enhanced by exoskeleton training, which provides task-specific, repetitive movements necessary to restore function in individuals with SCI. One study that focused on gait training combined with upper limb and trunk strength training reported that there were no participants in functional class A (slight gait impairment) prior to training, but by the end of the programme, there were 13 participants in this class and that by the completion of the training programme, no participants were in class C (severe gait limitation) compared to 18 participants prior to the intervention, and five more participants advanced to functional class B (moderate gait limitation) (Echemendía del Valle et al. 2023). These outcomes highlight the importance of targeting both lower and upper body muscle groups, as the harmony between them is crucial for gait stability and posture in SCI individuals. Additionally, the Walking Index for Spinal Cord Injury (WISCI) scores improved, which indicates an overall improvement in gait abilities and quality of life for those with an SCI (Echemendía del Valle et al. 2023).

Gait, the manner and process of walking, is a complex motor skill learned and essential for movement (Jankovic 2015). An SCI can partially or completely affect one’s ability to walk. Interventions that improve gait parameters can help restore mobility, enhance functional independence and improve overall quality of life (Jankovic 2015).

### Functional capacity

Five out of the 16 studies evaluated functional capacity variables as outcome measures of the study intervention (Amatachaya et al. 2019; Khurana et al. 2017; Martinez et al. 2018; Nithiatthawanon et al. 2020). Following treadmill training, three participants experienced an improvement in their LEMS, while four participants showed a decline and three exhibited no change. In contrast, after multimodal training, three participants improved their LEMS, three deteriorated and three remained unchanged (Martinez et al. 2018). These results suggest that the multimodal exercise rehabilitation programme, which combined balance exercises with advanced upper extremity exercises, did not provide any additional benefit compared with body weight-supported treadmill training (Martinez et al. 2018). Bodyweight shifting and lower limb loading exercises, with and without overground walking and external feedback as an intervention, as well as dual-task and single-task obstacle walking training, led to significant improvements in five-times sit-to-stand test (FTSST) measurements (Amatachaya et al. 2019; Nithiatthawanon et al. 2020). One study showed that virtual reality-based balance training improved balance and upper body function in people with paraplegia (Khurana et al. 2017).

Functional capacity refers to the extent of one’s ability to perform necessary activities and tasks (Patterson & Mausbach 2010). An SCI disrupts the motor and sensory pathways within the spinal cord, leading to a substantial decline in an individual’s functional abilities. Consequently, it is essential to identify the most effective interventions for enhancing functional capacity (Barranco et al. 2024).

### Impact on secondary complications

Three studies evaluated the effect of intervention on SCI-related secondary complications (Kim et al. 2015; Lambach et al. 2020; Ryan et al. 2013). One study reported that two participants with diabetes showed improved insulin sensitivity, and four participants demonstrated increased insulin sensitivity after training with electrically generated resistance exercises at home (Ryan et al. 2013). However, the small sample size makes it challenging to generalise these findings. This result correlates with findings from an indoor hand-bike training programme, which showed a significant decrease in fasting insulin levels post-intervention (Kim et al. 2015). Because of individuals with SCI often having a less active lifestyle, this finding is of significance because an increased insulin sensitivity reduces the risk of developing metabolic disorders, such as type 2 diabetes (Cragg et al. 2013). Both home-based electrically induced resistance exercise training and indoor hand-bike training showed a significant increase in high-density lipoprotein (HDL)-cholesterol levels post-intervention (Kim et al. 2015; Ryan et al. 2013), which further contributes to an improved metabolic profile. One study used FES rowing as an intervention and reported that most participants showed reduced bone loss in the tibia after 30 sessions, with minimal to no loss observed after 60–90 sessions (Lambach et al. 2020). Maintaining healthy bone density in individuals with an SCI is crucial, as low bone density can lead to fragility fractures, which may lead to further complications such as increased medical costs, hospital stays and reduced independence (Lambach et al. 2020). Reduced mobility and weight-bearing activity in SCI individuals often lead to rapid bone loss, especially in the lower limbs. This decline in BMD increases the risk of fractures, particularly in areas like the femur and tibia, which are less subject to mechanical loading post-injury (Sadowsky, Mingioni & Zinski 2020). Effective strategies to prevent bone loss include modalities such as weight-bearing exercises, resistance training and functional FES. Studies have shown that FES, which uses electrical impulses to stimulate paralysed muscles, can help maintain or even improve BMD by mimicking the mechanical loading that bones experience during movement. Early intervention is crucial for individuals with complete motor paralysis, as the first year is when the most bone loss occurs (Sadowsky et al. 2020).

The SCI population has shown greater intramuscular fat levels, which are associated with muscle atrophy, and both are linked to glucose intolerance, metabolic syndrome, dyslipidaemia, cardiovascular disease and osteoporosis (Cragg et al. 2013). Many of these secondary complications seen in individuals with SCI are brought to light by decreased levels of physical activity as paresis and paralysis result in a lack of opportunities for physical activity (Cragg et al. 2013). This highlights the importance of engaging in physical activity or exercise to combat such secondary complications.

## Implications and recommendations

### Study limitations

Although this scoping review presents promising data on the use of various exercise interventions to enhance the rehabilitation process after SCI, the evidence within this area of rehabilitation varies. The heterogeneity of the sample groups in terms of sample size, gender and completeness of injury makes it difficult to draw conclusions about the effectiveness of an intervention for any individual with an SCI.

### Future research recommendations

To progress the field of SCI rehabilitation, a few areas require further research. Longitudinal studies should be prioritised to analyse sustained effects of various exercise interventions among individuals with SCI, providing insights into the long-term benefits and potential challenges of different rehabilitation strategies. Additionally, future studies should aim to include larger and more homogeneous samples, as diverse sample demographics make it difficult to draw definitive conclusions about the efficacy of certain interventions across different demographics, such as gender, age and injury severity.

Gender-specific outcomes should be analysed, accounting for potential physiological differences between men and women that may influence their responses to exercise interventions, thereby allowing for more specific and effective rehabilitation programmes.

The use of emerging technologies, such as virtual reality and advanced exoskeletons, in rehabilitation programmes should be explored further. Additionally, more research is needed to understand the psychosocial outcomes of exercise interventions, as evidence in this area is lacking.

## Conclusion

The findings of this scoping review highlight the several rehabilitation intervention methods available to individuals with spinal cord injuries, especially those with lesion levels ranging from C5 to T12. The outcomes suggest a cardinal importance for an individual approach to therapy. Functional electrical stimulation and robotic-assisted gait training were shown to have the most significant improvements in muscle strength and aerobic capacity during early-phase rehabilitation. Functional electrical stimulation, specifically when combined with resistance training or rowing, improved muscle size, composition and metabolism, all of which are crucial during the early stages of recovery. The most effective exercise modalities for enhancing cardiovascular fitness, muscular endurance and functional mobility during late-phase rehabilitation were found to be upper extremity exercises, including hand-bike training, ergometric training and overground walking. This review highlights that no single intervention is universally effective for all aspects of SCI rehabilitation, as the nature of SCI rehabilitation requires an individualised approach. As physical rehabilitation therapists, understanding the limitations while considering the patient’s goals and needs will allow for more effective rehabilitation, enabling us to optimise rehabilitation outcomes across all stages of SCI rehabilitation.

## References

[CIT0001] Akkurt, H., Karapolat, H.U., Kirazli, Y. & Kose, T., 2017, ‘The effects of upper extremity aerobic exercise in patients with spinal cord injury: A randomised controlled study’, *European Journal of Physical and Rehabilitation Medicine* 53(2), 219–227. 10.23736/S1973-9087.16.03804-1 27824234

[CIT0002] Alizadeh, A., Dyck, S.M. & Karimi-Abdolrezaee, S., 2019, ‘Traumatic spinal cord injury: An overview of pathophysiology, models and acute injury mechanisms’, *Frontiers in Neurology* 10, 282. 10.3389/fneur.2019.00282 30967837 PMC6439316

[CIT0003] Amatachaya, S., Srisim, K., Arrayawichanon, P., Thaweewannakij, T. & Amatachaya, P., 2019, ‘Dual-task obstacle crossing training could immediately improve ability to control a complex motor task and cognitive activity in chronic ambulatory individuals with spinal cord injury’, *Topics in Spinal Cord Injury Rehabilitation* 25(3), 260–270. 10.1310/sci18-00038 31548793 PMC6743749

[CIT0004] Anjum, A., Yazid, M.D., Fauzi Daud, M., Idris, J., Ng, A.M.H., Selvi Naicker, A. et al., 2020, ‘Spinal cord injury: Pathophysiology, multimolecular interactions, and underlying recovery mechanisms’, *International Journal of Molecular Sciences* 21(20), 7533. 10.3390/ijms21207533 33066029 PMC7589539

[CIT0005] Barranco, L., Reis, K., Vigário, P., Sales, R. & Costa, L., 2024, ‘Eight weeks of functional training improves functional capacity in individuals with spinal cord injury’, *Brazilian Journal of Physical Therapy* 28(Suppl 1), 100831. 10.1016/j.bjpt.2024.100831

[CIT0006] Berlowitz, D.J., Wadsworth, B. & Ross, J., 2016, ‘Respiratory problems and management in people with spinal cord injury’, *Breathe (Sheffield, England)* 12(4), 328–340. 10.1183/20734735.012616 28270863 PMC5335574

[CIT0007] Bresnahan, J.J., Farkas, G.J., Clasey, J.L., Yates, J.W. & Gater, D.R., 2019, ‘Arm crank ergometry improves cardiovascular disease risk factors and community mobility independent of body composition in high motor complete spinal cord injury’, *The Journal of Spinal Cord Medicine* 42(3), 272–280. 10.1080/10790268.2017.1412562 29334345 PMC6522950

[CIT0008] Cragg, J.J., Noonan, V.K., Dvorak, M., Krassioukov, A., Mancini, G.B. & Borisoff, J.F., 2013, ‘Spinal cord injury and type 2 diabetes: Results from a population health survey’, *Neurology* 81(21), 1864–1868. 10.1212/01.wnl.0000436074.98534.6e 24153440 PMC3821709

[CIT0009] Echemendía del Valle, A., Bender del Busto, J.E., Sentmanat Belisón, A., Cuenca-Zaldívar, J.N., Martínez-Pozas, O., Martínez-Lozano, P. et al., 2023, ‘Effects of a gait training program on spinal cord injury patients: A single-group prospective cohort study’, *Journal of Clinical Medicine* 12(23), 7208. 10.3390/jcm12237208 38068259 PMC10707500

[CIT0010] Farahani, M.F., Khankeh, H.R., Hosseini, M., Dalvandi, A. & NorouziTabrizi, K., 2021, ‘Exploring facilitators of regaining autonomy in people with spinal cord injury: A qualitative study’, *Iranian Journal of Nursing and Midwifery Research* 26(2), 154–161. 10.4103/ijnmr.IJNMR_25_20 34036064 PMC8132859

[CIT0011] Frison, V.B., Lanferdini, F.J., Geremia, J.M., De Oliveira, C.B., Radaelli, R., Netto, C.A. et al., 2019, ‘Effect of corporal suspension and pendulum exercises on neuromuscular properties and functionality in patients with medullar thoracic injury’, *Clinical Biomechanics (Bristol, Avon)* 63, 214–220. 10.1016/j.clinbiomech.2019.02.012 30952032

[CIT0012] Gagnon, D.H., Escalona, M.J., Vermette, M., Carvalho, L.P., Karelis, A.D., Duclos, C. et al., 2018, ‘Locomotor training using an overground robotic exoskeleton in long-term manual wheelchair users with a chronic spinal cord injury living in the community: Lessons learned from a feasibility study in terms of recruitment, attendance, learnability, performance and safety’, *Journal of NeuroEngineering and Rehabilitation* 15(1), 12. 10.1186/s12984-018-0354-2 29490678 PMC5831695

[CIT0013] Gaspar, R., Padula, N., Freitas, T.B., De Oliveira, J.P.J. & Torriani-Pasin, C., 2019, ‘Physical exercise for individuals with spinal cord injury: Systematic review based on the international classification of functioning, disability, and health’, *Journal of Sport Rehabilitation* 28(5), 505–516. 10.1123/jsr.2017-0185 30300056

[CIT0014] Gorgey, A.S. & Dudley, G.A., 2007, ‘Skeletal muscle atrophy and increased intramuscular fat after incomplete spinal cord injury’, *Spinal Cord* 45(4), 304–309. 10.1038/sj.sc.3101968 16940987

[CIT0015] Hodgkiss, D.D., Bhangu, G.S., Lunny, C., Jutzeler, C.R., Chiou, S.Y., Walter, M. et al., 2023, ‘Exercise and aerobic capacity in individuals with spinal cord injury: A systematic review with meta-analysis and meta-regression’, *PLoS Medicine* 20(11), e1004082. 10.1371/journal.pmed.1004082 38011304 PMC10712898

[CIT0016] Ilyas, S., Tariq, I., Anwar, K., Arshad, H. & Butt, M.W., 2023, ‘Effects of upper limb ergometer on pulmonary functions among spinal cord injury patients’, *Physiotherapy Quarterly* 31(4), 15–20. 10.5114/pq.2023.116840

[CIT0017] Jankovic, J., 2015, ‘Gait disorders’, Neurologic Clinics 33(1), 249–268. 10.1016/j.ncl.2014.09.007 25432732

[CIT0018] Kesiktaş, F.N., Kaşıkçıoğlu, E., Paker, N., Bayraktar, B., Karan, A., Ketenci, A. et al., 2021, ‘Comparison of the functional and cardiovascular effects of home-based versus supervised hospital circuit training exercises in male wheelchair users with chronic paraplegia’, *Turkish Journal of Physical Medicine and Rehabilitation* 67(3), 275–282. 10.5606/tftrd.2021.6533 34870113 PMC8606995

[CIT0019] Khurana, M., Walia, S. & Noohu, M.M., 2017, ‘Study on the effectiveness of virtual reality game-based training on balance and functional performance in individuals with paraplegia’, *Topics in Spinal Cord Injury Rehabilitation* 23(3), 263–270. 10.1310/sci16-00003 29339902 PMC5562034

[CIT0020] Kim, D.I., Lee, H., Lee, B.S., Kim, J. & Jeon, J.Y., 2015, ‘Effects of a 6-week indoor hand-bike exercise program on health and fitness levels in people with spinal cord injury: A randomized controlled trial study’, *Archives of Physical Medicine and Rehabilitation* 96(11), 2033–2040. 10.1016/j.apmr.2015.07.010 26254953

[CIT0021] Lambach, R.L., Stafford, N.E., Kolesar, J.A., Kiratli, B.J., Creasey, G.H., Gibbons, R.S. et al., 2020, ‘Bone changes in the lower limbs from participation in an FES rowing exercise program implemented within two years after traumatic spinal cord injury’, *The Journal of Spinal Cord Medicine* 43(3), 306–314. 10.1080/10790268.2018.1544879 30475172 PMC7241570

[CIT0022] Maher, J.L., McMillan, D.W. & Nash, M.S., 2017, ‘Exercise and health-related risks of physical deconditioning after spinal cord injury’, *Topics in Spinal Cord Injury Rehabilitation* 23(3), 175–187. 10.1310/sci2303-175 29339894 PMC5562026

[CIT0023] Martinez, S.A., Nguyen, N.D., Bailey, E., Doyle-Green, D., Hauser, H.A., Handrakis, J.P., et al., 2018, ‘Multimodal cortical and subcortical exercise compared with treadmill training for spinal cord injury’, *PLoS One* 13(8), e0202130. 10.1371/journal.pone.0202130 30092092 PMC6084979

[CIT0024] Nithiatthawanon, T., Amatachaya, P., Thaweewannakij, T., Manimmanakorn, N., Sooknuan, T. & Amatachaya, S., 2020, ‘Immediate effects of lower limb loading exercise during stepping with and without augmented loading feedback on mobility of ambulatory individuals with spinal cord injury: A single-blinded, randomized, cross-over trial’, *Spinal Cord* 58(12), 1301–1309. 10.1038/s41393-020-0498-3 32632173

[CIT0025] Park, J.H., Kim, H.S., Jang, S.H., Hyun, D.J., Park, S.I., Yoon, J. et al., 2021, ‘Cardiorespiratory responses to 10 weeks of exoskeleton-assisted overground walking training in chronic nonambulatory patients with spinal cord injury’, *Sensors* 21(15), 5022. 10.3390/s21155022 34372258 PMC8347087

[CIT0026] Patterson, T.L. & Mausbach, B.T., 2010, ‘Measurement of functional capacity: A new approach to understanding functional differences and real-world behavioral adaptation in those with mental illness’, *Annual Review of Clinical Psychology* 6, 139–154. 10.1146/annurev.clinpsy.121208.131339 PMC3160788 20334554

[CIT0027] Pramodhyakul, N., Amatachaya, P., Sooknuan, T., Arayawichanon, P. & Amatachaya, S., 2016, ‘Visuotemporal cues clinically improved walking ability of ambulatory patients with spinal cord injury within 5 days’, *The Journal of Spinal Cord Medicine* 39(4), 405–411. 10.1179/2045772315Y.0000000058 26507118 PMC5102287

[CIT0028] Richardson, A., Samaranayaka, A., Sullivan, M. & Derrett, S., 2021, ‘Secondary health conditions and disability among people with spinal cord injury: A prospective cohort study’, *The Journal of Spinal Cord Medicine* 44(1), 19–28. 10.1080/10790268.2019.1581392 30882288 PMC7919890

[CIT0029] Ryan, T.E., Brizendine, J.T., Backus, D. & McCully, K.K., 2013, ‘Electrically induced resistance training in individuals with motor complete spinal cord injury’, *Archives of Physical Medicine and Rehabilitation* 94(11), 2166–2173. 10.1016/j.apmr.2013.06.016 23816921

[CIT0030] Sadowsky, C.L., Mingioni, N. & Zinski, J., 2020, ‘A primary care provider’s guide to bone health in spinal cord-related paralysis’, *Topics in Spinal Cord Injury Rehabilitation* 26(2), 128–133. 10.46292/sci2602-128 32760192 PMC7384544

[CIT0031] Santos, L.V., Pereira, E.T., Reguera-García, M.M., Oliveira, C.E.P. & Moreira, O.C., 2022, ‘Resistance training and muscle strength in people with spinal cord injury: A systematic review and meta-analysis’, *Journal of Bodywork and Movement Therapies* 29, 154–160. 10.1016/j.jbmt.2021.09.031 35248264

[CIT0032] Sargent, L., Smitherman, J., Sorenson, M., Brown, R. & Starkweather, A., 2023, ‘Cognitive and physical impairment in spinal cord injury: A scoping review and call for new understanding’, *The Journal of Spinal Cord Medicine* 46(3), 343–366. 10.1080/10790268.2022.2134634 36441038 PMC10114976

[CIT0033] Taylor, J.A., Picard, G., Porter, A., Morse, L.R., Pronovost, M.F. & Deley, G., 2014, ‘Hybrid functional electrical stimulation exercise training alters the relationship between spinal cord injury level and aerobic capacity’, *Archives of Physical Medicine and Rehabilitation* 95(11), 2172–2179. 10.1016/j.apmr.2014.07.412 25152170 PMC4252514

[CIT0034] Vermaak, C., Ferreira, S., Terblanche, E. & Derman, W., 2022, ‘Physical activity promotion in persons with spinal cord injuries: Barriers and facilitators in low-resource communities’, *African Journal of Disability* 11(1), 988–996. https://doi/full/10.4102/ajod.v11i0.988 35812772 10.4102/ajod.v11i0.988 PMC9257716

